# UvPomt, an O-Methyltransferase Interacting with UvMAT1-1-3, for Regulating Growth, Stress Tolerance, and Virulence in *Ustilaginoidea virens* †

**DOI:** 10.3390/jof11060426

**Published:** 2025-05-31

**Authors:** Zhi Li, Junjie Yu, Mina Yu, Huijuan Cao, Tianqiao Song, Shuchen Wang, Zhongqiang Qi, Yan Du, Xiayan Pan, Yongfeng Liu

**Affiliations:** 1 College of Plant Protection, Nanjing Agricultural University, Nanjing 210095, China; 2021202006@stu.njau.edu.cn; 2 Institute of Plant Protection, Jiangsu Academy of Agricultural Sciences, Nanjing 210014, China; jjyu@jaas.ac.cn (J.Y.); 20130030@jaas.ac.cn (M.Y.); caohuijuan@jaas.ac.cn (H.C.); tqsong123456@163.com (T.S.); 20230089@jaas.ac.cn (S.W.); 20130019@jaas.ac.cn (Z.Q.); 20130011@jaas.ac.cn (Y.D.)

**Keywords:** rice false smut, *Ustilaginoidea virens*, fungal pathogenicity, fungi sexual reproduction, mating-type gene, O-methyltransferase

## Abstract

Rice false smut (RFS), caused by *Ustilaginoidea virens* (teleomorph: *Villosiclava virens*), is a devastating fungal disease that severely impacts global rice production by reducing both yield and grain quality. While the mating-type gene *UvMAT1-1-3* is known to regulate both sexual and asexual reproduction in *U. virens,* its regulatory mechanism remains unclear. In this study, an interacting protein of UvMAT1-1-3, a putative O-methyltransferase (UvPomt), was identified using yeast two-hybrid screening, and its interaction was further confirmed by co-localization microscopy. A quantitative reverse transcription PCR (qRT-PCR) analysis showed a significant up-regulation of *UvPomt* expression during the early infection stage of *U. virens*. Functional characterization revealed that Δ*UvPomt* mutants exhibited reduced fungal pathogenicity, vegetative growth, conidial production, and stress adaptation. Furthermore, a Western blot analysis revealed that the UvMAT1-1-3 protein level was reduced in Δ*UvPomt* mutants, whereas the UvPomt protein level was elevated in Δ*UvMAT1-1-3* mutants. Taken together, these findings suggest a potential reciprocal regulation between UvPomt and UvMAT1-1-3. Understanding *UvPomt*’s function could provide a potential molecular target for controlling RFS disease.

## 1. Introduction

The ascomycete fungus *Ustilaginoidea virens* (teleomorph: *Villosiclava virens*) is the causal agent of rice false smut (RFS), a globally destructive floral disease affecting *Oryza sativa* [1]. RFS not only causes significant yield losses but also threatens food safety through the production of mycotoxins, particularly ustiloxins and ustilaginoidins, which accumulate in false smut balls [2,3]. Recent studies have documented an increasing incidence of RFS in major rice-producing regions worldwide [4]. This trend highlights the urgent need to develop targeted strategies for the sustainable management of this devastating disease.

The life cycle of *U. virens* undergoes two distinct reproductive phases: asexual propagation via chlamydospores and sexual reproduction through sclerotia [1]. The asexual reproductive cycle of *U. virens* involves chlamydospore formation on false smut balls. Two functional spore types emerge: primary chlamydospores exhibit immediate infectivity, directly invading florets of late-maturing cultivars within the current season, while dormant chlamydospores persist over winter through seedborne transmission or environmental dispersal. These overwintering propagules initiate mycelial germination during panicle initiation under warm, humid irrigation conditions, subsequently developing secondary conidia that serve as the primary inoculum for floral infection [5]. During the sexual life cycle of *V. virens*, fertile sclerotia form on the surface of the false smut balls in rice spikelets infected by two mating-type strains during late autumn. Overwinter, fertile sclerotia germinate under suitable conditions and produce numerous ascospores, which serve as the infection sources in the disease cycle [6].

The sexual reproduction in ascomycetous fungi is controlled by a conserved genetic regulatory mechanism centered on the mating-type (*MAT*) locus, which encodes master transcription regulators controlling reproductive compatibility [7]. Fungal sexual reproduction is classified into three distinct types based on *MAT* gene organization and nuclear distribution. In homothallism, a single nucleus contains both *MAT1-1* and *MAT1-2* alleles, enabling self-fertility without a mating partner. For instance, *Aspergillus nidulans* produces a fused *MAT1-1*/*MAT1-2* transcript for autonomous reproduction [8]. In contrast, in heterothallism, *MAT1-1* and *MAT1-2* alleles are distributed among different fungi, requiring mating between compatible strains. For example, *Neurospora crassa* uses pheromone signaling for partner recognition, completing the process of sexual reproduction [9]. Two nuclei with different *MAT* alleles coexist in the same cell in pseudohomothallism, enabling both selfing and outcrossing [10]. The expression of mating-type (*MAT*) genes is regulated by multiple factors, including MADS-box transcription factors and histone acylation modifications [11,12].

*U. virens* is a bipolar heterothallic ascomycete whose sexual reproduction is coordinately regulated by *MAT1-1* and *MAT1-2* idiomorphs [13]. The *MAT1-1* idiomorph includes four genes: *UvMAT1-1-1*, *UvMAT1-1-2*, *UvMAT1-1-3,* and truncated *UvMAT1-2-1*. The *MAT1-2* idiomorph includes *UvMAT1-2-1* and *UvMAT1-2-8*. The functional characterization of mating-type genes in the *MAT1-1* idiomorph revealed that *UvMAT1-1-2* and *UvMAT1-1-3* are essential for sclerotia formation, while *UvMAT1-1-1*, although dispensable for sclerotia development, is required for subsequent stroma formation. Both *UvMAT1-1-2* and *UvMAT1-1-3* are critical for rice pathogenicity. Beyond sexual reproduction, these genes regulate asexual processes including mycelial growth, conidiation, and stress responses. Importantly, the interaction between UvMAT1-1-1 and UvMAT1-1-3 suggests their coordinated regulation through a genetic network. In addition, UvMAT1-1-1 and UvMAT1-1-2 are localized in the nucleus and cytoplasm, respectively, while UvMAT1-1-3 is localized in both compartments, implying a unique regulatory role during sexual reproduction. However, the specific regulatory mechanisms of *UvMAT1-1-3* remain unclear [14,15].

Caffeic acid O-methyltransferase (COMT) is a key S-adenosylmethionine (SAM)-dependent methyltransferase in the phenylpropanoid pathway, which regulates lignin and flavonoid biosynthesis through substrate-specific methylation in plant metabolic cascades [16]. In plants, COMT mediates various stress responses. The ectopic expression of *LbOMT* in *Lilium brownii* and *Arabidopsis thaliana* confers enhanced resistance to *Botrytis cinerea* by promoting lignin deposition and phytoalexin biosynthesis [17]. While, *Arabidopsis thaliana* overexpressing *ZmCOMT* exhibits increased melatonin secretion and improved salt tolerance [18]. In contrast to its well-documented roles in plants, the function of COMT in phytopathogens remains poorly characterized [19].

In this study, we characterized a putative O-methyltransferase (GenBank: QUC16060.1, designated as UvPomt) as an interacting protein of UvMAT1-1-3 using yeast two-hybrid screening. A qRT-PCR analysis revealed its up-regulation in the early infection of *U. virens*. The CRISPR/Cas9-mediated knockout of *UvPomt* demonstrated that *UvPomt* plays a role in fungal morphogenesis, pathogenicity, and stress adaptation. Furthermore, we verified that UvPomt directly interacts with UvMAT1-1-3 and reciprocally regulates their protein levels. These findings suggest that *UvPomt* act as a potential molecular target for controlling rice false smut disease.

## 2. Materials and Methods

### 2.1. Strains and Culture Conditions

The *U. virens* strain P1 [15] was used as the wild-type strain for subsequent strain construction. For strain activation, 40 µL aliquots of glycerol stock from −80 °C were aseptically inoculated onto potato sucrose agar (PSA: 200 g/L potato, 20 g/L sucrose, and 15 g/L agar) plates, followed by incubation at 28 °C for 5 days to ensure robust mycelial growth. For routine culturing, strains were grown on the following media: PSA medium for general fungal maintenance and yeast extract tryptone agar (YTA: 1 g/L yeast extract, 1 g/L tryptone, 10 g/L sucrose, and 15 g/L agar) medium for phenotypic characterization. All fungal cultures were incubated at 28 °C in darkness for 10–20 days. For pathogenicity assays, susceptible rice (*Oryza sativa* L. cv. Liangyoupei9, LYP9) seedlings were cultivated in a controlled warm house (at 28 °C, 95% humidity, and with 12 h/12 h light/dark cycle). Yeast strain Y2H Gold was cultured on yeast extract peptone glucose agar (YPDA: 10 g/L yeast extract, 20 g/L peptone, 20 g/L glucose, 0.3 g/L adenine hemi sulfate, and 15 g/L agar) medium at 30 °C. *Agrobacterium tumefaciens* strain AGL1 was used for *Agrobacterium tumefaciens*-mediated transformation (ATMT). *Escherichia coli* DH5α was used for plasmid construction [20,21].

### 2.2. Phylogenetic Analysis of Homologous Proteins

Homologous protein sequence of UvPomt was downloaded from the National Center for Biotechnology Information (NCBI, http://www.ncbi.nlm.nih.gov/, accessed on 14 January 2025) database, and their accession numbers are as follows: *Ustilaginoidea virens* (XP_042993733.1), *Metarhizium anisopliae* (KAF5127807.1), *Metarhizium acridum* (XP 065976325.1), *Epichloë festucae* (QPH05175.1), *Fusarium solani* (XP_046134121.1), *Fusarium oxysporum* (KAK2700284.1), *Fusarium fujikuroi* (KLP16479.1), *Pyricularia grisea* (XP_030984444.1), *Epichloë bromicola* (GAB0134975.1), *Purpreocillium lavendulum* (KAJ6443186.1), *Fusarium falciforme* (KAJ4206499.1), *Fusarium albosuccineum* (KAF4455525.1), *Purpureocillium lilacinum* (OAQ82954.1), and *Fusarium venenatum* (XP_025590785.1). Phylogenetic analysis was conducted using the neighbor-joining method implemented in MEGA 11 (Molecular Evolutionary Genetics Analysis). Branch support was assessed with 1000 bootstrap replicates to ensure robust statistical evaluation of tree topology.

The O-methyltransferase PCMT domains from six fungal species were subjected to comparative analysis: *Ustilaginoidea virens* (UvPomt), *Metarhizium anisopliae* (MaPomt), *Epichloë festucae* (EfOMT), *Fusarium sacchari* (FsOMT), *Fusarium oxysporum* (FoOMT), and *Pyricularia grisea* (PgOMT). Phylogenetic analysis was performed using the neighbor-joining method implemented in MEGA 11. Branch support was assessed with 1000 bootstrap replicates to ensure robust statistical evaluation of tree topology. The conserved domain of UvPomt was predicted by SMART (http://smart.embl-heidelberg.de/, accessed on 15 January 2025) with default parameters.

### 2.3. Construction of UvPomt Knockout and Complementation Strains

To generate *UvPomt* knockout mutants, we deleted *UvPomt* (Gene ID: *Uv8b_00301*) via homologous recombination using CRISPR/Cas9 (clustered regularly interspaced short palindromic repeats) system [22]. The *UvPomt* gene replacement plasmid was generated by amplifying 1 kb upstream and 1 kb downstream flanking sequences from the wild-type strain P1 genomic DNA, followed by ligation with the hygromycin resistance cassette into pMD19-T-vector (Takara, Kusatsu, Japan; Cat. #6013). In parallel, the sgRNA expression vector pCas9-*UvPomt* was constructed for CRISPR/Cas9-mediated editing. Both pMD19*-UvPomt* and pCas9*-UvPomt* were co-transformed into wild-type strain P1 via polyethylene glycol (PEG)-mediated transformation [22]. Hygromycin-resistant transformants were screened by PCR assays and further by DNA sequencing to confirm homologous recombination (Figure S1). Genome DNA was extracted using DNA extract kit (CWBIO, Nanjing, China, CW0531M), which employs a silica-membrane column-based workflow. Sample concentration and purity were measured using a NanoDrop spectrophotometer (A260/A280 ≥ 1.8).

To functionally validate UvPomt, the complementation vector was constructed by ligating the full-length *UvPomt* ORF, along with its 2 kb native promoter and 0.7 kb terminator region, into the BamHI/EcoRI sites of the pKO1 vector. The resulting pKO1-*UvPomt* construct was introduced into the Δ*UvPomt-247* via Agrobacterium tumefaciens-mediated transformation (ATMT) [23]. G418 resistance transformants were confirmed by RT-PCR at the mRNA level (Figure S1).

To generate UvPomt-GFP fusion strain, we amplified the ORF region of UvPomt and cloned it into pKD1-GFP, then transformed pKD1-UvPomt-GFP into P1 by ATMT and obtained GFP-tagging strains after 15-day transformation. Hygromycin-resistant transformants were confirmed by microscopic observation (Carl Zeiss AG, Jena, Germany) and Western blotting of anti-GFP antibody.

The primers used above are listed in Supplementary Table S1.

### 2.4. Phenotypic Analysis of U. virens Strains

To assess stress sensitivity of *UvPomt* in *U. virens*, wild-type strain P1, gene deletion mutants (∆*UvPomt-247* and ∆*UvPomt-279*), and complement strain (C∆*UvPomt-1*) were cultured on PSA medium for 7 days. Mycelial plugs (with 5 mm diameter) of each strain were then transferred to YTA plates supplemented with 0.04 mg/mL CFW, 0.03% H_2_O_2_, 200.00 mM Congo red, 0.20 M NaCl, and 0.60 M sorbitol according to our previous study [24]. Following 14 days of incubation at 28 °C, colony diameters were measured. Growth inhibition rate was calculated as: Inhibition rate (%) = [(Control diameter − Treated diameter)/Control diameter] × 100. For mycelial growth assay, each *U. virens* strain was incubated for 14 days in the dark at 28 °C in YTA medium, after which the colony diameters were measured. For conidiation assays, mycelial plugs (with 5 mm diameter) from each strain were transferred into 50 mL of liquid YT medium in 250 mL flasks and cultured at 28 °C for 6 days (150 rpm). All experiments were performed three times with three technical replicates [25]. Mycelium growth and stress sensitivity tests were performed at the same time.

### 2.5. Pathogenicity and Plant Infection Assays

The wild-type strain P1, ∆*UvPomt* mutants (∆*UvPomt-247* and ∆*UvPomt-279*), and complement strain (C∆*UvPomt-1*) were cultured in PSB liquid medium at 28 °C with shaking at 160 rpm for 6 days. Conidial suspensions (containing both conidia and mycelia) were adjusted into 1 × 10^6^ conidia/mL, and 2 mL suspensions were injected into rice panicles (LYP9) at the booting stage. Inoculated plants were maintained at 28 °C and 95% relative humidity for 4 weeks. Three independent biological experiments were performed, with 10 technical replicates (individual panicle) per strain per experiment [26]. Disease severity was quantified by counting rice false smut balls on each infected panicle.

### 2.6. RNA Manipulations and qRT-PCR

Rice spikelets inoculated with wild-type P1 were collected at 0, 1, 3, 5, and 7 day post-inoculation (dpi). Total RNA was extracted using the RNAprep Pure Plant Kit (CWBIO, Nanjing, China, CW0598S) following the manufacturer’s protocol. RNA concentration and purity were determined using a NanoDrop spectrophotometer (Thermo Fisher Scientific, Waltham, MA, USA) with A260/A280 ratio ≥ 2.0. For cDNA synthesis, 1 µg total RNA was reverse-transcribed using HiScript III All-in-one RT SuperMix (Vazyme, Nanjing, China, #R223-01) in a 20 µL reaction volume. qRT-PCR was performed using ChamQ Universal SYBR qPCR Master Mix (Vazyme, Nanjing, China, #Q711-02) in 20 µL volume to detect the transcription level of *UvPomt* during infection stage. Relative mRNA amounts were calculated using the 2^−ΔΔCT^ method [27]. *UvPomt* expression during infection stage was normalized to the *U. virens* housekeeping gene *UvTubulin* (*Uv8b_05680*). The 0 dpi timepoint was designated as control, with all subsequent timepoints expressed as fold-changes relative to this baseline.

Total RNA of the wild-type P1, ∆*UvPomt-247*, and ∆*UvMAT1-1-3-40* mutant were extracted from 5-day-old vegetative hyphae. RNA extraction and cDNA reverse transcription method were performed as described above. *UvMAT1-1-3* expression in P1 and Δ*UvPomt-247* and *UvPomt* expression in P1 and ∆*UvMAT1-1-3-40* were normalized to the fungal reference gene *UvTubulin*.

The specific PCR primers used above are listed in Supplementary Table S1.

### 2.7. Yeast Two-Hybrid Assay

Protein–protein interactions were verified by yeast two-hybrid assay. The ORF region of *UvPomt* was cloned into pGADT7 vector, and the ORF region of *UvMAT1-1-3* was cloned into pGBKT7 using the ClonExpress II One Step Cloning Kit (Vazyme, Nanjing, China, #C113). The pairs of yeast two-hybrid plasmids were co-transformed into Y2H Gold via the LiAc carrier DNA/PEG method (Hua Yueyang, Beijing, China). To verify the certainty of the interaction by Y2H, we co-transformed plasmids pGADT7-*UvMAT1-1-1* and pGBKT7-*UvMAT1-1-3* to serve as the positive control, while pGADT7 and pGBKT7 were used as negative control [15]. Transformants grew on SD/-Leu/-Trp and SD/-Ade/-His/-Leu/-Trp medium for 3–5 days at 30 °C. The experiments were independently repeated three times to confirm the results.

### 2.8. Western Blotting Assay

The transformants used for experiment were shaken at 28 °C, 170 rpm for 5 dpi. Approximately 100 mg mycelia were suspended with NP40 (Beyotime, P0013F, Shanghai, China) and 10 µL of PMSF (Beyotime, ST505, Shanghai, China). A 20 µL total protein sample was separated on 12% SDS-PAGE gels and then transferred onto a polyvinylidene fluoride (PVDF) membrane with a BioRad electroblotting apparatus. Subsequent experiments were performed with detection by anti-GFP primary antibody (1:3000, Abmart, M20004, Shanghai, China), anti-Actin (1:2000, Zhongding Biotech, 10011, Nanjing, China), and anti-mouse secondary antibody (1:3000, Abmart, M21001, Shanghai, China). All antibodies were diluted with PBS buffer. The signal was detected by ECL chemiluminescence solution for 3–5 min. The *Actin* gene was used as the internal reference.

### 2.9. Statistical Analysis

The experimental design incorporated triplicate biological replicates with quintuple technical repetitions per treatment group. The significant value of the differences in all analyses was evaluated using the least significant difference test with the Duncan’s test in SPSS 17.0. Results are presented as mean ± SD, with significance thresholds established at *p* < 0.05 and *p* < 0.01 for differential expression analysis.

## 3. Results

### 3.1. Identification of UvPomt in U. virens by Y2H

**Figure 1:**
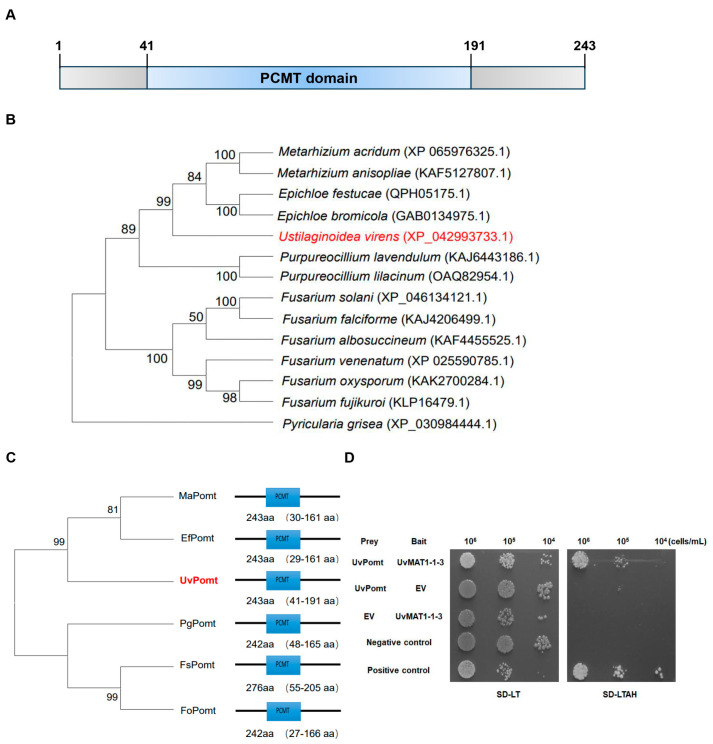
Sequence and phylogenetic analysis of UvPomt in *U. virens*. (**A**) Domain architecture of UvPomt showing a conserved domain: PCMT domain (41-191 aa). (**B**) Phylogenetic analysis of O-methyltransferase homologs in fungal species. (**C**) Phylogenetic reconstruction of fungal O-methyltransferases reveals evolutionary conservation of PCMT domains linked to pathogenic adaptation. The UvPomt in *U. virens* is marked in red. (**D**) Yeast two-hybrid assay was to examine the interaction between UvPomt and UvMAT1-1-3. The resulting yeast transformants were assayed for growth on SD/-Leu/-Trp and SD/-Leu/-Trp/-Ade/-His/medium.

To explore the regulatory network of UvMAT1-1-3, an important mating-type protein governing both sexual and asexual reproduction in *U. virens*, we performed Y2H screening using the full-length UvMAT1-1-3 as a bait against a cDNA library derived from mixed-stage fungal cultures [26]. A putative O-methyltransferase (designated as UvPomt; GenBank accession: XP_042993733.1) was identified to interact with UvMAT1-1-3 through screening. Domain structure annotation via the SMART database revealed that the protein UvPomt contains a PCMT domain (Pfam: PF01135; InterPro: IPR000278) encompassing residues 41-191 (Figure 1A). The phylogenetic tree showed that UvPomt shares a high degree of sequence similarity with its homolog in *Metarhizium anisopliae*, but a lower degree of similarity with that in the plant pathogen *Fusarium solani* (Figure 1B,C). The interaction between UvPomt and UvMAT1-1-3 was further confirmed by the Y2H assay. As shown in Figure 1D, UvPomt demonstrated a directly physical interaction with UvMAT1-1-3.

### 3.2. UvPomt Is Essential for U. virens Virulence

**Figure 2:**
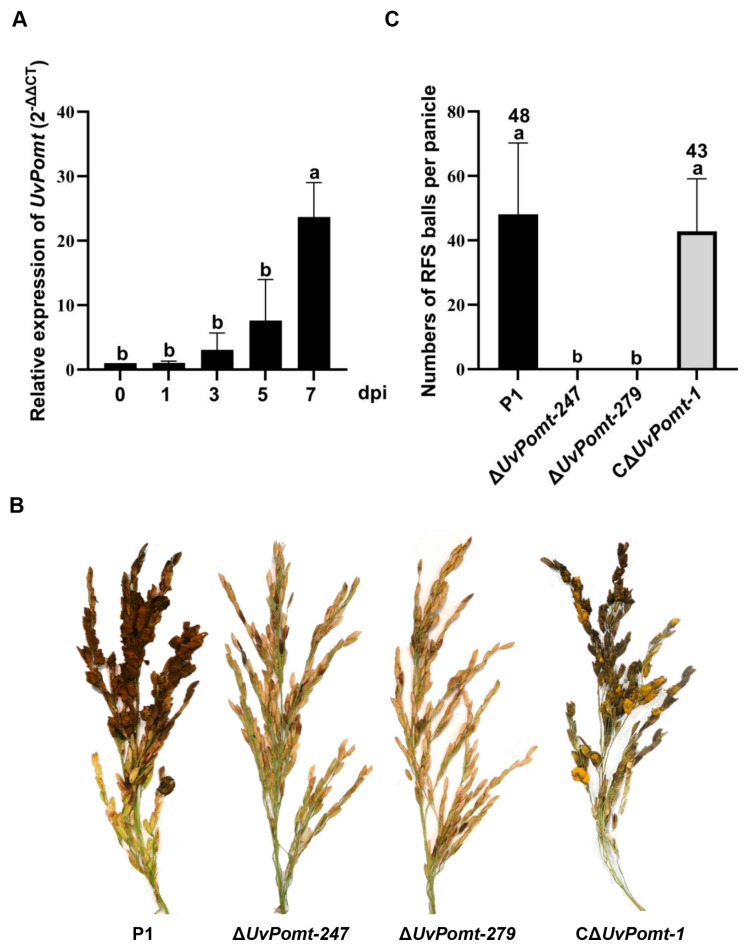
Roles of *UvPomt* in *U. virens* virulence. (**A**) qRT-PCR assay results evaluating the expression pattern of *UvPomt* during the infection stage (at 0, 1, 3, 5, and 7 dpi). Data are presented as mean ± SD. Experiment was repeated two times with similar results (Duncan’s test, *p* < 0.01). (**B**) Symptoms of rice panicles inoculated with P1, the Δ*UvPomt* mutants (∆*UvPomt*-*247,* ∆*UvPomt*-*279*), and the complemented strain CΔ*UvPomt-1* at 30 dpi on susceptible rice cultivar LYP9. (**C**) Statistical analysis of false smut balls per panicle. Data are presented as mean ± SD. Different letters indicate significant differences (Duncan’s test, *p* < 0.01).

To determine whether the screened interaction protein UvPomt plays an important role in the infection stage of *U. virens*, qRT-PCR was performed to determine its gene expression during different infection stages. The results showed that the gene expression of *UvPomt* was significantly up-regulated at 7 dpi compared with 0 dpi, suggesting that UvPomt may play a critical role in the infection process of *U. virens* (Figure 2A).

To further characterize the virulence of *UvPomt* in *U. virens*, we generated three ∆*UvPomt* mutants (∆*UvPomt*-*247*, ∆*UvPomt-279*, and ∆*UvPomt-323*) and one complemented strain (C∆*UvPomt-1*) (Figure S1). The wild-type strain P1 and complemented strain C∆*UvPomt-1* showed approximately 40–50 rice false smut balls per panicle, while the ∆*UvPomt* mutants ∆*UvPomt*-*247* and ∆*UvPomt*-*279* completely lost their virulence (Figure 2B,C). These results demonstrate that *UvPomt* is essential for the pathogenicity of *U. virens*.

### 3.3. UvPomt Is Involved in Hyphae and Conidiation Capacity

To further assess the role of *UvPomt* in fungal development, we assessed the mycelial growth and conidiation of P1, the Δ*UvPomt* mutants (Δ*UvPomt*-*247* and Δ*UvPomt*-*279*), and the complemented strain (CΔ*UvPomt*-*1*) on YTA medium.

**Figure 3:**
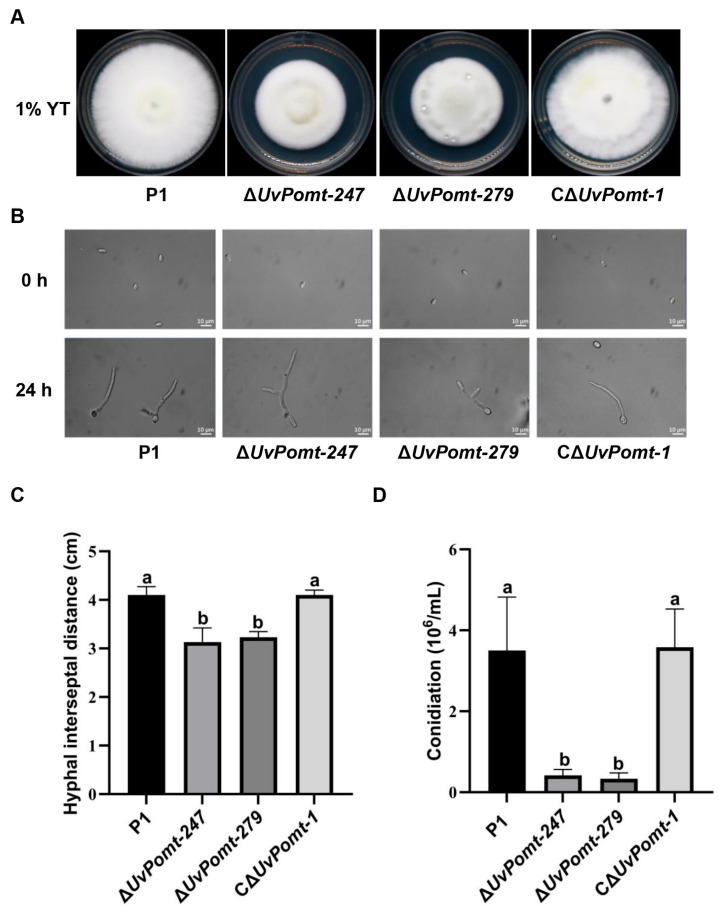
*UvPomt* is required for vegetative growth and conidial production in *U. virens*. (**A**) Colony morphology of the wild-type strain P1, the ∆*UvPomt* mutants, and the complemented strain *C*∆*UvPomt*-*1*. Representative images from the same experimental batch are also shown in Figure 4A. (**B**) Conidial morphology of P1, the ∆*UvPomt* mutants, and the complemented strain. (**C**) Colony diameters of tested strains. Data are presented as mean ± SD of three biological replicates. Different letters indicate significant differences (Duncan’s test, *p* < 0.01). (**D**) Conidiation capacity of tested strains. Data are presented as mean ± SD of three biological replicates. Different letters indicate significant differences (Duncan’s test, *p* < 0.01).

After the 14-day incubation on YTA plates, the Δ*UvPomt*-*247* and Δ*UvPomt*-*279* exhibited colony diameters that were 23.6% and 21.1% smaller compared to those of wild-type P1 (Figure 3A,C). The genetic complementation strain (CΔ*UvPomt*-*1*) fully restored vegetative growth, confirming the phenotype’s linkage to *UvPomt* deletion. These results indicate that Δ*UvPomt* mutants exhibit defective vegetative growth.

Meanwhile, the conidiation capacity was significantly impaired in the Δ*UvPomt* mutants, with Δ*UvPomt*-*247* [(0.42 ± 0.14) × 10^6^ conidia/mL] and Δ*UvPomt*-*279* [(0.33 ± 0.14) × 10^6^ conidia/mL] producing only 12.0% and 9.4% of the levels of P1 [(3.50 ± 1.33) × 10^6^ conidia/mL], respectively (Figure 3B,D). The Δ*UvPomt* mutants showed a significant decrease in conidiation. These results suggest that *UvPomt* is essential for conidiation.

### 3.4. UvPomt’s Role in Response to Environmental Stresses

**Figure 4:**
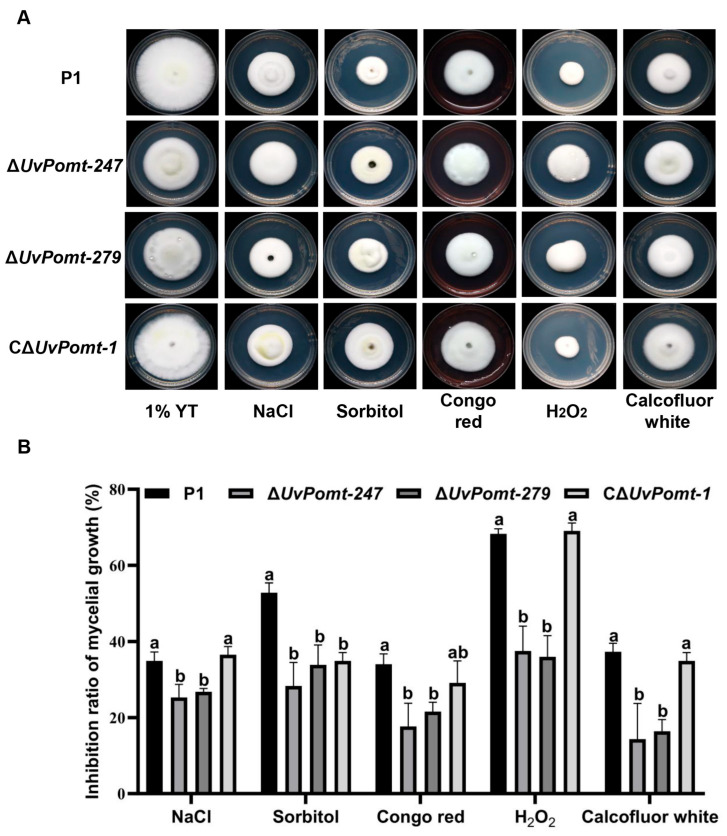
*UvPomt* is involved in stress responses in *U. virens*. (**A**) Colony morphology of P1, the ∆*UvPomt* mutants, and the complemented strain C∆*UvPomt*-*1* on YTA including 0.20 M NaCl, 0.60 M sorbitol, 200.00 mM Congo red (CR), 0.03% H_2_O_2_, and 0.04 mg/mL Calcofluor-white (CFW) in the dark for 14 days. (**B**) Column diagram showing the growth inhibition of tested strains under various stress conditions. Data are presented as mean ± SD of three biological replicates. Different letters indicate significant differences (Duncan’s test, *p* < 0.01).

To determine whether *UvPomt* regulates the stress adaptation of *U. virens*, we assessed the growth of wild-type P1, the Δ*UvPomt* mutants (Δ*UvPomt-247* and Δ*UvPomt-279*), and the complemented strain (CΔ*UvPomt-1*) on YTA plates supplemented with stressors: H_2_O_2_ (oxidative stress), Calcofluor-white (CFW, cell-wall-damaging agent), and Congo red (CR, cell wall stressor), NaCl, and Sorbitol (osmotic stress). The Δ*UvPomt* mutants exhibited significantly higher growth rates under the tested stress conditions compared with P1 and the complemented strain (Figure 4). These results suggest that *UvPomt* deletion increases tolerance to the tested stressors.

### 3.5. UvPomt Affects the Protein Expression Level of UvMAT1-1-3 in U. virens

**Figure 5:**
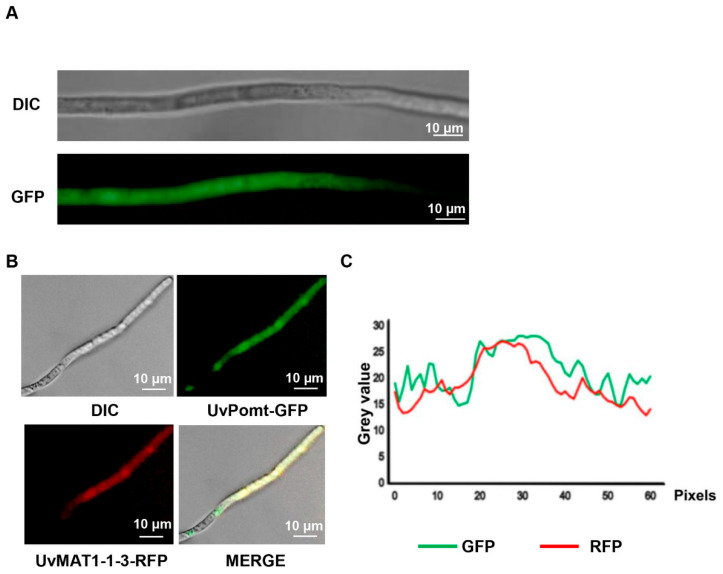
Sub-cellular localization of UvPomt in mycelia. (**A**) The sub-cellular localization of UvPomt in the hyphae of *U. virens*. Vegetative hyphae of the transformant expressing UvPomt-GFP were observed under confocal microscopy. (**B**) Sub-cellular localization of UvPomt and UvMAT1-1-3 in mycelia. Vegetative hyphae of transformant expressing UvPomt-GFP and UvMAT1-1-3-RFP were observed under fluorescence microscopy. (**C**) Fluorescence intensity analysis of the transformant co-expressing UvPomt-GFP (green) and UvMAT1-1-3-RFP (red).

To determine the sub-cellular localization of UvPomt in *U. virens*, we generated the UvPomt-GFP fusion construct and expressed it in *U. virens*. A Western blotting analysis with anti-GFP antibody confirmed the expected molecular mass of the fusion protein (~50 kDa), which was consistent with the combined size of UvPomt and GFP (Figure S2). Confocal laser scanning microscopy (CLSM) revealed that UvPomt-GFP localized in both the cytoplasm and nucleus (Figure 5A), consistent with previous findings showing that UvMAT1-1-3 is localized in the cytoplasm and nucleus of *U. virens* [13]. To examine the spatial relationship between UvPomt and UvMAT1-1-3, we observed the sub-cellular location of P1 with pKD1-UvPomt-GFP (a GFP-tagged UvPomt construct) and pKD2-UvMAT1-1-3-RFP (an RFP-tagged UvMAT1-1-3 construct) under CLSM. The results showed that UvPomt and UvMAT1-1-3 localized in the hyphae of *U. virens* (Figure 5B,C).

**Figure 6:**
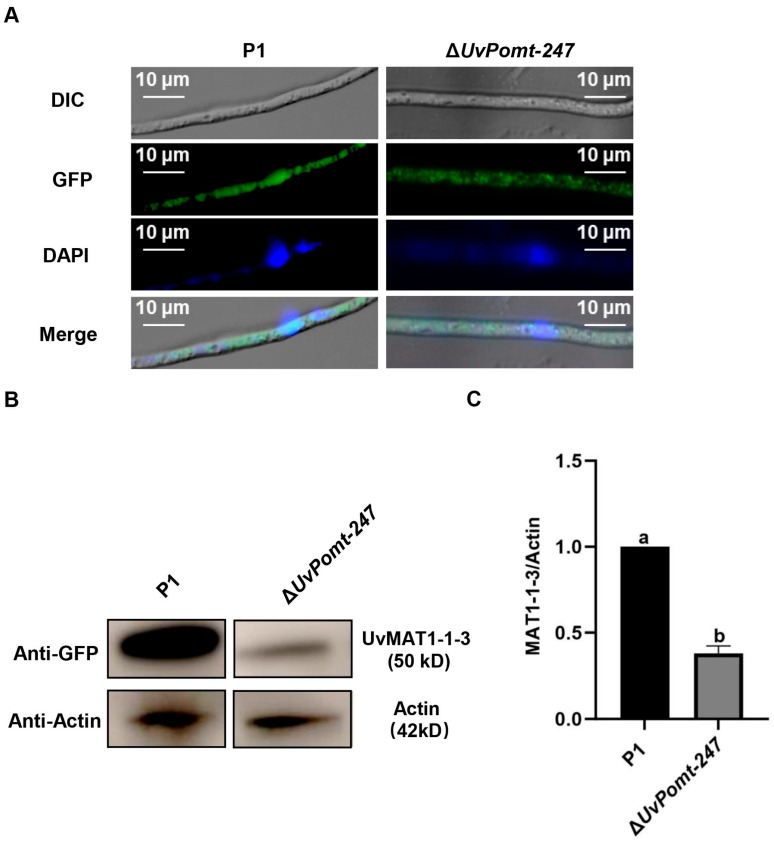
Regulation of UvMAT1-1-3 protein level by UvPomt. (**A**) Sub-cellular localization of UvMAT1-1-3-GFP in P1 and ∆*UvPomt*-*247* mutant. (**B**) Western blotting of UvMAT1-1-3 in P1 and ∆*UvPomt*-*247* mutant. (**C**) Relative UvMAT1-1-3 protein levels in P1 and ∆*UvPomt-247* mutant. The MAT1-1-3 protein level in P1 was set to 1.0. Data are presented as mean ± SD, based on three independent replicates. Different letters indicate significant differences (Duncan’s test, *p* < 0.01).

To elucidate the regulatory relationship between UvPomt and UvMAT1-1-3 in *U. virens*, we analyzed their sub-cellular localization, gene expression, and protein levels. As shown in Figure 6A, the deletion of *UvPomt* did not alter the localization pattern of UvMAT1-1-3. The qRT-PCR analysis showed that the deletion of *UvPomt* had no significant effect on *UvMAT1-1-3* gene expression (Figure S3). However, Western blotting demonstrated a significant reduction in UvMAT1-1-3 protein levels in the Δ*UvPomt*-247 mutant (Figure 6B,C). Intriguingly, UvPomt protein levels were up-regulated in the Δ*UvMAT1-1-3* mutant (Figure S4). These results suggest a reciprocal post-transcriptional regulation between UvPomt and UvMAT1-1-3, regulating their protein abundance in *U. virens*.

### 3.6. UvPomt Affects On the Gene Expression level of Pathogenicity and Sexual Reproduction-Related Genes in U. virens

**Figure 7:**
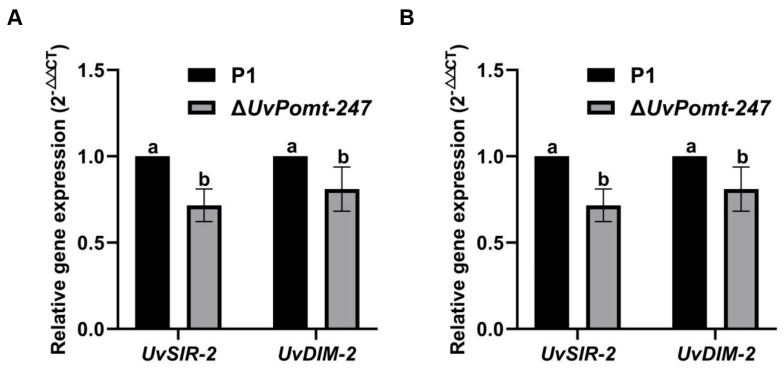
The gene transcription of pathogenicity and sexual-reproduction-related genes in Δ*UvPomt* mutant. (**A**) The gene expression levels of pathogenicity-related genes in the P1 and ∆*UvPomt*-*247* mutant. Data are presented as mean ± SD of three independent replicates. Different letters indicate significant differences (Duncan’s test, *p* < 0.01). (**B**) The gene expression levels of sexual-reproduction-related genes in the P1 and ∆*UvPomt*-*247* mutant. Data are presented as mean ± SD of three independent replicates. Different letters indicate significant differences (Duncan’s test, *p* < 0.01).

Since Δ*UvPomt* mutants resulted in the loss of pathogenicity, we investigated whether *UvPomt* regulates pathogenicity-related genes. A qRT-PCR analysis revealed a significant down-regulation of two key epigenetic regulators, DNA methyltransferase *UvDIM-2* and histone deacetylase *UvSIR-2*, in the Δ*UvPomt* mutant compared to the wild-type strain P1 (Figure 7A). Notably, the expression of these genes was similarly suppressed in the ∆*UvMAT1-1-3* mutant (RNA-seq data) [15]. These findings suggest UvPomt and UvMAT1-1-3 may share similar regulatory pathways in controlling pathogenicity.

As *UvMAT1-1-3* is important for the sexual reproduction of *U. virens* [15], we investigated genes involved in sexual reproduction regulated by *UvPomt*. We performed qRT-PCR to detect the gene expression of *UvPRE1* and *UvPRE2.* Our data showed that *UvPRE1* and *UvPRE2* were down-regulated in the Δ*UvPomt* mutant (Figure 7B). These results suggest that *UvPomt* may regulate sexual reproduction in *U. virens*.

## 4. Discussion

The mating-type gene *UvMAT1-1-3* plays a crucial role in *U. virens*, regulating both vegetative growth and reproductive development [15]. In this study, Y2H screening identified a putative O-methyltransferase, UvPomt, as an interacting protein of UvMAT1-1-3 (Figure 1A). qRT-PCR analysis revealed that it is significantly up-regulated during early infection, suggesting its involvement in pathogenic processes. Phenotypic characterization exhibited that *UvPomt* plays an important role in hyphal growth, conidiation, fungal pathogenicity, and stress adaptation, implying its importance in fungal development. Furthermore, we found a post-transcriptional reciprocal regulation between UvPomt and UvMAT1-1-3, suggesting a potential functional dependency between them.

Phylogenetic analysis revealed that UvPomt forms a distinct clade with orthologous proteins from the entomopathogenic fungus *Metarhizium anisopliae* and the endophytic fungus *Epichloë festucae*. The high degree of sequence conservation among these species suggests that UvPomt likely plays an important and evolutionarily conserved role in fungal cross-kingdom adaptation. Notably, the observed sequence divergence in *Fusarium solani* homologs implies potential functional specialization within this protein family across different fungal lineages, which may reflect adaptations to specific ecological niches or host environments (Figure 1B,C).

In *Lilium brownii*, caffeine-O-methyltransferase (COMT) enhances resistance against the pathogen *Botrytis cinerea* by promoting lignin biosynthesis and detoxifying phenolic compounds [27]. However, the role of O-methyltransferases in fungal pathogenicity remains unclear. In this study, we demonstrate that UvPomt, an O-methyltransferase homolog in *U. virens*, is critical for the full virulence of *U. virens* (Figure 2). *U. virens* establishes infection by hijacking host nutrients to form false smut balls [1]. Previous studies have shown that disrupting key virulence factors, such as Δ*UvCOM1*, permits early nutrient acquisition but blocks smut ball formation [28], while septin deletion eradicates floral infection entirely [26]. UvPomt diverges functionally from plant COMT orthologs. In maize, *ZmCCoAOMT2* enhances apoplastic immunity by mediating lignin deposition for cell wall fortification, thereby restricting pathogen invasion [29], whereas its homologs in *Fusarium graminearum* have evolved distinct catalytic roles in methylating aromatic intermediates during the biosynthesis of mycotoxins [19]. This divergence hints at UvPomt’s potential role in producing virulence-associated metabolites in *U. virens*. Y2H assays confirmed a direct interaction between UvPomt and UvMAT1-1-3 (Figure 1D), suggesting their potential functional cooperation in regulating *U. virens* pathogenicity.

Including the role in pathogenicity, *UvPomt* plays an important function in fungal growth, conidiation, and stress adaptation. Δ*UvPomt* mutants exhibited reduced vegetative growth and conidial production compared to wild-type P1 and the complemented strain (CΔ*UvPomt-1*) (Figure 3), the results exhibited that *UvPomt* is a key regulator of vegetative growth and conidial production. The growth defects of *UvPomt*, coupled with its role in oxidative and osmotic stress tolerance (Figure 4), suggests that *UvPomt* mediates a balance between growth and stress adaptation, a strategy likely critical for *U. virens* to thrive in the dynamic rice floret microenvironment. In *Capsicum annuum*, *CaCOMT13* and *CaCOMT46* help plants cope with low-temperature conditions and salt stress accumulation, underscoring their conserved role in stress adaptation [28]. In contrast, our study uncovers a distinct role in *U. virens*: *UvPomt* deletion reduced fungal sensitivity to osmotic, cell-wall-disrupting, and oxidative stress (Figure 4), suggesting that *UvPomt* suppresses abiotic stress responses in *U. virens*. O-methyltransferases exhibit functional divergence in plants and fungi, showing that they have evolved distinct mechanisms to develop specialized metabolic functions. For instance, such enzymes in plants are primarily responsible for reinforcing the cell walls [30], whereas similar enzymes in fungi are involved in producing toxins or adapting to the infection environment [19]. Notably, the Δ*UvMAT1-1-3* mutant displayed similar stress sensitivity attenuation [15], suggesting that *UvPomt* may regulate abiotic stress adaptation through UvMAT1-1-3-dependent pathways.

Confocal microscopy analysis revealed that UvPomt and UvMAT1-1-3 showed co-localization in *U. virens* (Figure 5B,C), indicating a compartment-specific regulation of UvMAT1-1-3 activity. This interaction may link nuclear transcriptional control with cytoplasmic post-translational signaling. This mechanism contrasts with the exclusively nuclear SmMCM1 and SmSMTA-1 interaction in *Sordaria macrospora*, in which chromatin remodeling drives sexual development [11]. In filamentous ascomycetes, mating-type loci serve as master regulators of both sexual and asexual reproduction, and their roles in governing mating-type gene expression have been extensively characterized. For instance, studies in *Ustilago maydis* revealed that the cAMP-PKA signaling pathway exerts control over mating-type genes through deacetylation of the histone deacetylase UmHOS2 [12]. Our findings of UvPomt as an interacting protein of UvMAT1-1-3 suggests a potential regulatory mechanism in *U. virens*, raising intriguing questions about its interplay with established mating-type signaling. Notably, our findings reveal a critical regulatory relationship between UvPomt and the mating-type transcription factor UvMAT1-1-3. In the *ΔUvPomt* strain, the UvMAT1-1-3 protein level was significantly reduced. This reduction occurred despite unaltered transcript levels, implying that *UvPomt* mediates the post-transcriptional regulation of UvMAT1-1-3, possibly through regulating protein stability or ubiquitination-mediated degradation. Furthermore, UvMAT1-1-3 was shown to co-regulate UvPomt protein levels, indicating a mutual regulation between these proteins (Figure 6).

Additionally, *UvMAT1-1-3* positively regulates the expression of the histone deacetylase *UvSIR-2* and the heterochromatin protein *UvDIM-2* (Figure 7A), a regulatory pattern that diminished the hypo-virulent phenotype of Δ*UvMAT1-1-3* mutants [30,31], which is consistent with the pathogenic phenotype of Δ*UvPomt* mutants, suggesting that UvPomt may participate in UvMAT1-1-3 during fungal development.

*UvMAT1-1-3* is important for sexual reproduction in *U. virens*, and considering that successful sexual reproduction requires infection by two compatible mating strains, however, because of non-pathogenic of Δ*UvPomt* mutants, we were unable to obtain sclerotia under the experimental conditions. To investigate the role of *UvPomt* in sexual reproduction, we performed a qRT-PCR analysis to examine its expression patterns during the pre-sexual stages (*UvPRE1* and *UvPRE2*) (Figure 7B). Our results showed the down-regulation of *UvPRE1* and *UvPRE2* expression in *U. virens*, suggesting its potential role in the sexual reproduction cycle of *U. virens* [32].

UvPomt regulates the protein abundance of UvMAT1-1-3, suggesting a potential link between *UvPomt* activity and mating competence in *U. virens*. Above results exhibited UvPomt interacting with UvMAT1-1-3, not only providing new insights into the regulatory network governing fungal development but also explaining the potential role of *UvPomt* in mediating sexual reproduction.

## 5. Conclusions

In summary, this study demonstrates that *UvPomt* is a key pathogenesis-related gene in *U. virens*, regulating vegetative growth, conidiation, pathogenicity, and stress tolerance. Biochemical analyses demonstrated that UvPomt reciprocally regulates the protein level of UvMAT1-1-3, and their direct interaction was confirmed by Y2H assays and a co-localization analysis. Future work should employ domain truncation, transcriptomics, and time-course co-immunoprecipitation to unravel the UvPomt-UvMAT1-1-3 mechanism, potentially guiding antifungal strategies targeting fungal development and stress adaption.

## Data Availability

The original contributions presented in this study are included in the article/Supplementary Materials. Further inquiries can be directed to the corresponding authors.

## Supplementary Materials

The following supporting information can be downloaded at: https://www.mdpi.com/article/10.3390/jof11060426/s1. Figure S1. Targeted knockout and complementation of *UvPomt* in *U. virens*. (A) PCR detection of ∆*UvPomt* mutants (∆*UvPomt*-*247*, ∆*UvPomt*-*279* and ∆*UvPomt*-*323*). The wild-type P1 served as negative control. (B) Schematic diagram of the construction of replacement vector. (C) RT-PCR analysis of validating the expression of *UvPomt* gene in ∆*UvPomt* mutants (∆*UvPomt*-*247*, ∆*UvPomt*-*279* and ∆*UvPomt-323*), the wild-type P1 and the complemented strain *C*∆*UvPomt-1*. Figure S2. UvPomt-GFP protein is shown by Western blotting. Lane 1: total protein extract from GFP-expressing strain P1 (negative control); lane 2: total protein extract from UvPomt-GFP-expressing strain P1. Figure S3. The transcription of *UvMAT1-1-3* in ∆*UvPomt* mutant. The gene expression levels of *UvMAT1-1-3* in the P1 and ∆*UvPomt-247* mutant. Data are represented as mean ± SD based on three independent replicates (ns, *p* > 0.05, Duncan’s test). Figure S4. Regulation of *UvPomt* gene expression and the encoding protein level by UvMAT1-1-3. (A) The gene expression levels of UvPomt in the P1 and ∆*UvMAT1-1-3-40* mutant. Data are presented as mean ± SD based on three independent replicates (ns, *p* > 0.05, Duncan’s test). (B) Western blotting of UvPomt in the P1 and ∆*UvMAT1-1-3-40* mutant. lane 1: total protein extract from P1; Lane 2: total protein extract from ∆*UvMAT1-1-3-40*. (C) Quantitative analysis of UvPomt protein levels in the P1 and ∆*UvMAT1-1-3-40* mutant. The UvPomt protein level in P1 was set to 1.0. Data are presented as mean ± SD based on three independent replicates. Different letters indicate significant differences (Duncan’s test, *p* < 0.05). Table S1. Primers used in this study. Table S2. Stains used in this study. Table S3. Plasmids used in this study.

## References

[1] Sun W.X., Fan J., Fang A.F., Li Y.J., Tariqjaveed M., Li D.Y., Hu D.W., Wang W.M. (2020). *Ustilaginoidea virens*: Insights into an emerging rice pathogen. Annu. Rev. Phytopathol..

[2] Li Y.J., Wang M., Liu Z.H., Zhang K., Cui F.H. (2020). Towards understanding the biosynthetic pathway for *Ustilaginoidea* mycotoxins in *Ustilaginoidea virens*. Environ. Microbiol..

[3] Long Z.Y., Wang P.Y., Yu Q.H., Wang B., Li D.Y., Yang C., Liu L., Duan G.H., Sun W.X. (2024). The histone deacetylase UvHOS2 regulates vegetative growth, conidiation, ustilaginoidin synthesis, and pathogenicity in *Ustilaginoidea virens*. Phytopathol. Res..

[4] Liu X.Y., Matsumoto H., Lv T.X., Zhan C.F., Fang H.D., Pan Q.Q., Xu H.R., Fan X.Y., Chu T.Y., Chen S.L. (2023). Phyllosphere microbiome induces host metabolic defence against rice false-smut disease. Nat. Microbiol..

[5] Song J.H., Wei W., Lv B., Lin Y., Yin W.X., Peng Y.L., Schnabel G., Huang J.B., Jiang D.H., Luo C.X. (2016). Rice false smut fungus hijacks the rice nutrient supply by blocking and mimicking the fertilization of rice ovary. Environ. Microbiol..

[6] Deng Q.D. (2015). The Function of Sclerotia in the Life History of Rice False Smut Pathogen. Master’s Thesis.

[7] Whittle C.A., Nygren K., Johannesson H. (2011). Consequences of reproductive mode on genome evolution in fungi. Fungal Genet. Biol..

[8] Pyrzak W., Miller K.Y., Miller B.L. (2008). Mating type protein Mat1-2 from asexual *Aspergillus fumigatus* drives sexual reproduction in fertile *Aspergillus nidulans*. Eukaryot. Cell.

[9] Liu H.Q., Li Y., Chen D.P., Qi Z.M., Wang Q.H., Wang J.H., Jiang C., Xu J.R. (2017). A-to-I RNA editing is developmentally regulated and generally adaptive for sexual reproduction in *Neurospora crassa*. Proc. Natl. Acad. Sci. USA.

[10] Svedberg J., Nygren K., Menkis A., James T.Y., Wik L., Stajich J.E., Johannesson H. (2010). Conflict between reproductive gene trees and species phylogeny among heterothallic and pseudohomothallic members of the filamentous ascomycete genus *Neurospora*. Fungal Genet. Biol..

[11] Nolting N., Pöggele S.A. (2006). MADS box protein interacts with a mating-type protein and is required for fruiting body development in the homothallic ascomycete *Sordaria macrospora*. Eukaryot. Cell.

[12] Elías-Villalobos A., Fernández-Álvarez A., Moreno-Sánchez I., Helmlinger D., Ibeas J.I. (2015). The Hos2 histone deacetylase controls *Ustilago maydis* virulence through direct regulation of mating-type genes. PLoS Pathog..

[13] Yu J.J., Sun W.X., Yu M.N., Yin X.L., Meng X.K., Zhao J., Huang L., Liu Y.F. (2015). Characterization of mating-type loci in rice false smut fungus *Villosiclava virens*. FEMS Microbiol. Lett..

[14] Yong M.L., Yu J.J., Pan X.Y., Yu M.N., Cao H.J., Song T.Q., Qi Z.Q., Du Y., Zhang R.S., Yin X.L. (2020). Two mating-type genes MAT1-1-1 and MAT1-1-2 with significant functions in conidiation, stress response, sexual development, and pathogenicity of rice false smut fungus *Villosiclava virens*. Curr. Genet..

[15] Yong M.L., Yu J.J., Pan X.Y., Yu M.N., Cao H.J., Qi Z.Q., Du Y., Zhang R.S., Song T.Q., Yin X.L. (2020). MAT1-1-3, a mating type gene in *Villosiclava virens*, is required for fruiting bodies and sclerotia formation, asexual development and pathogenicity. Front. Microbiol..

[16] Yang W.J., Du Y.T., Zhou Y.B., Chen J., Xu Z.S., Ma Y.Z., Chen M., Min D.H. (2019). Overexpression of *TaCOMT* improves melatonin production and enhances drought tolerance in transgenic *Arabidopsis*. Int. J. Mol. Sci..

[17] Liu X. (2024). Cloning and Functional Analysis of Caffeine-Induced *LrCOMT* Gene by *Botrytis cinerea* in Lily of the Minjiang River. Master’s Thesis.

[18] Zhang L., Zhao Y.Q., Fu J.F., Zhang Q., Ma C.Y., Li Y.Z., Zhang L., Wang G.G. (2023). Genome-wide identification and functional analysis of caffeic acid from maize. Fujian J. Agric. Sci..

[19] Ou P.P., He Q.L., Zhao Q. (2023). Structural diversification of natural substrates modified by the AurJ from *Fusarium graminearum*. Biochem. Biophys. Res. Commun..

[20] Xue M.Y., Hou X.W., Gu G., Dong J., Yang Y.L., Pan X.Q., Zhang X., Xu D., Lai D.W., Zhou L.G. (2024). Activation of ustilaginoidin biosynthesis Gene *uvpks1* in *Villosiclava virens* albino strain LN02 influences development, stress responses, and inhibition of rice seed germination. J. Fungi.

[21] Wang B., Duan G., Liu L., Long Z., Bai X., Ou M.M., Wang P.Y., Jiang D., Li D.Y., Sun W.X. (2024). UvHOS3-mediated histone deacetylation is essential for virulence and negatively regulates ustilaginoidin biosynthesis in *Ustilaginoidea virens*. Mol. Plant Pathol..

[22] Liu L., Wang B., Duan G.H., Wang J., Pan Z.Q., Ou M.M., Bai X.L., Wang P.Y., Zhao D., Nan N. (2023). Histone deacetylase UvHST2 is a global regulator of secondary metabolism in *Ustilaginoidea virens*. J. Agric. Food Chem..

[23] Qu J.S., Wang Y.F., Xia M.Z., Liu Y.R., Gu L.F., Zhou P., Du Y.L., Xu C.H., Wang R., Yin W.X. (2022). The bZIP transcription factor UvbZIP6 mediates fungal growth, stress response, and false smut formation in *Ustilaginoidea virens*. Phytopathol. Res..

[24] Xu Y.D., Wu S., Yu Z.M., Moeketsi E.K., Yang Z.X., Zhang Z.G., Zhang H.F. (2021). Transcription factor UvMsn2 is important for vegetative growth, conidiogenesis, stress response, mitochondrial morphology, and pathogenicity in the rice false smut fungus *Ustilaginoidea virens*. Phytopathol. Res..

[25] Chen X.Y., Li P.P., Liu H., Chen X.L., Huang J.B., Luo C.X., Li G.T., Hsiang T., Collinge D.B., Zheng L. (2021). A novel transcription factor UvCGBP1 regulates development and virulence of rice false smut fungus *Ustilaginoidea virens*. Virulence.

[26] Zhao J. (2015). Research on the Mating-Type Gene Expression Characteristics of *Ustilaginoidea virens* and Preliminary Study on MAT1-1-3 Interacting Proteins. Master’s Thesis.

[27] Gu Z.M., Li L.Z., Yin H.X. (2024). Genome-wide identification and expression analysis of caffeic acid *COMT* gene family in *Capsicum*. J. Plant Physiol..

[28] Chen X.Y., Hai D., Tang J.T., Liu H., Huang J.B., Luo C.X., Hsiang T., Zheng L. (2020). *UvCom1* is an important regulator required for development and infection in the rice false smut fungus *Ustilaginoidea virens*. Phytopathology.

[29] Yang Q., He Y.J., Kabahuma M., Chaya T., Kelly A., Borrego E., Bian Y., Kasmi F.E., Yang L., Teixeira P. (2017). A gene encoding maize caffeoyl-CoA confers quantitative resistance to multiple pathogens. Nat. Genet..

[30] Li H.M., Mo P.C., Zhang J., Xie Z.E., Liu X.Y., Chen H., Yang L.Y., Liu M.X., Zhang H.F., Wang P. (2023). Methionine biosynthesis enzyme MoMet2 is required for rice blast fungus pathogenicity by promoting virulence gene expression via reducing 5mC modification. PLoS Genet..

[31] Xu H.J., Ye M., Xia A.L., Jiang H., Huang P.P., Liu H.Q., Hou R., Wang Q.H., Li D.G., Xu J.R. (2023). The Fng3 ING protein regulates H3 acetylation and H4 deacetylation by interacting with two distinct histone-modifying complexes. New Phytol..

[32] Mayrhofer S., Weber J.M., Pöggeler S. (2006). Pheromones and pheromone receptors are required for proper sexual development in the homothallic ascomycete *Sordaria macrospora*. Genetics.

