# Supplementary material for: UvPomt, an O-Methyltransferase Interacting with UvMAT1-1-3, for Regulating Growth, Stress Tolerance, and Virulence in Ustilaginoidea virens
Source: J Fungi (Basel). 2025 May 31;11(6):426. doi: 10.3390/jof11060426 (PMC12194633; doi:10.3390/jof11060426)
Supplement: Supplementary file 1 [file jof-11-00426-s001.zip › supplement figure.pdf]

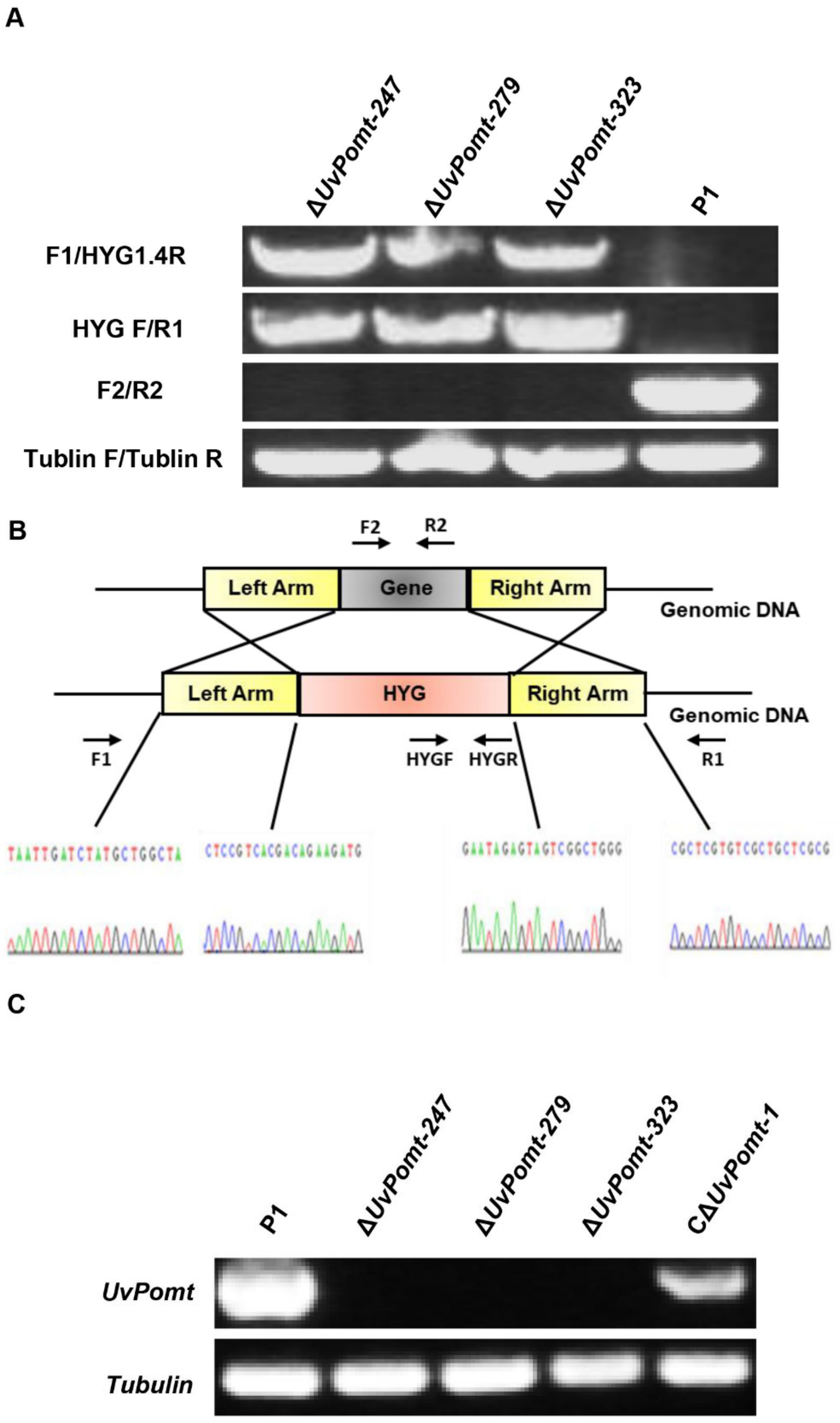

Figure S1. Targeted knockout and complementation of *UvPomt* in *U. virens*. (**A**) PCR detection of Δ*UvPomt* mutants (Δ*UvPomt-247*, Δ*UvPomt-279* and Δ*UvPomt-323*). The wild-type P1 served as negative control. (**B**) Schematic diagram of the construction of replacement vector. (**C**) RT-PCR analysis of validating the expression of *UvPomt* gene in Δ*UvPomt* mutants (Δ*UvPomt-247*, Δ*UvPomt-279* and Δ*UvPomt-323*), the wild-type P1 and the complemented strain CΔ*UvPomt-1*.

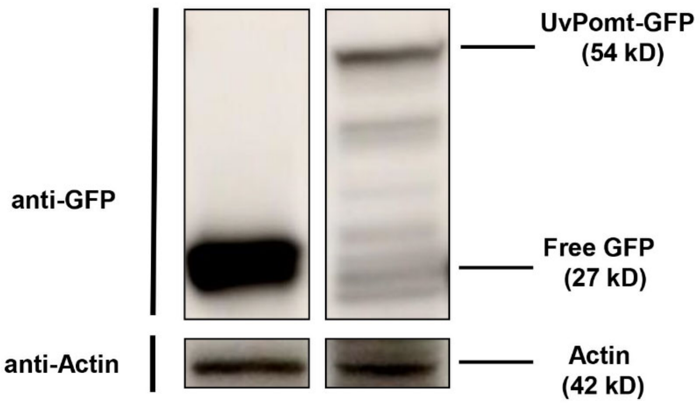

Figure S2. UvPomt-GFP protein is shown by Western blotting. Lane 1: total protein extract from GFP-expressing strain P1 (negative control); lane 2: total protein extract from UvPomt-GFP-expressing strain P1.

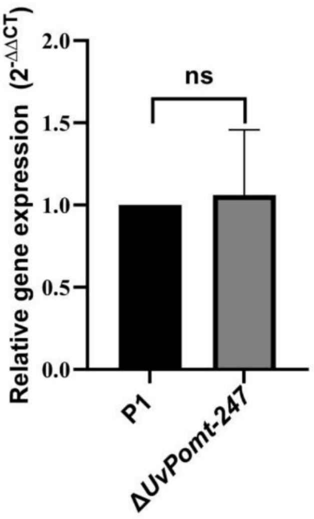

Figure S3. The transcription of *UvMAT1-1-3* in Δ*UvPomt* mutant. The gene expression levels of *UvMAT1-1-3* in the P1 and Δ*UvPomt-247* mutant. Data are represented as mean ± SD based on three independent replicates (ns, $p > 0.05$, Duncan's test).

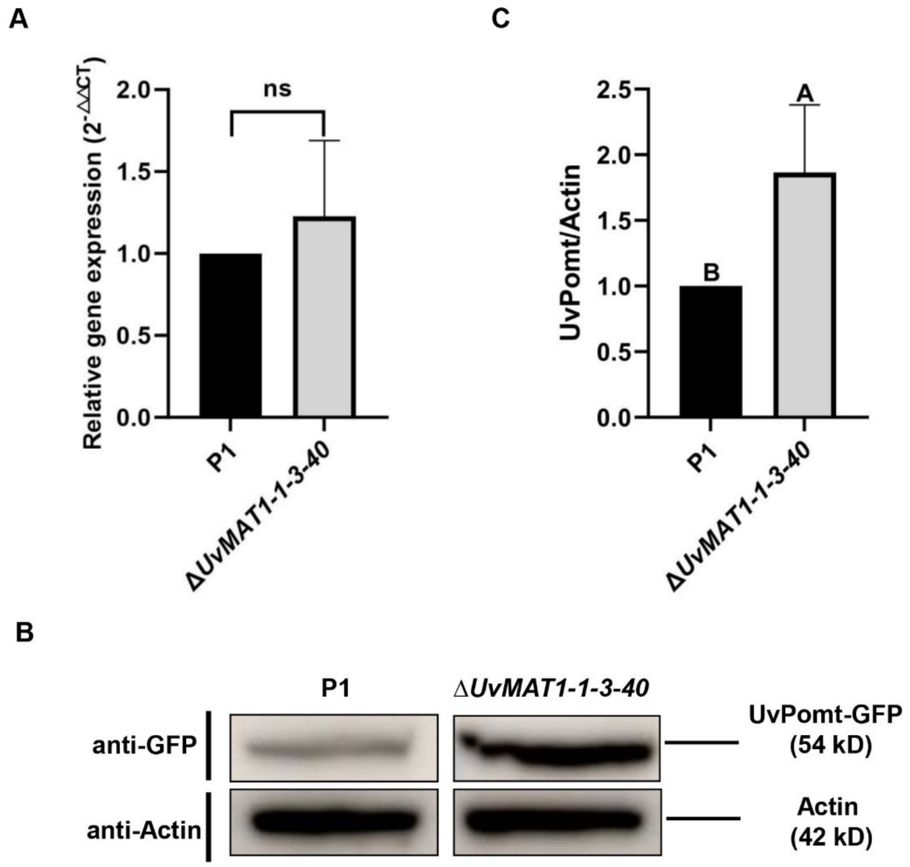

Figure S4. Regulation of *UvPomt* gene expression and the encoding protein level by UvMAT1-1-3. (**A**) The gene expression levels of *UvPomt* in the P1 and Δ*UvMAT1-1-3-40* mutant. Data are presented as mean ± SD based on three independent replicates (ns, $p > 0.05$, Duncan's test). (**B**) Western blotting of UvPomt in the P1 and Δ*UvMAT1-1-3-40* mutant. lane 1: total protein extract from P1; Lane 2: total protein extract from Δ*UvMAT1-1-3-40*. (**C**) Quantitative analysis of UvPomt protein levels in the P1 and Δ*UvMAT1-1-3-40* mutant. The UvPomt protein level in P1 was set to 1.0. Data are presented as mean ± SD based on three independent replicates. Different letters indicate significant differences (Duncan's test, $p < 0.05$).
